# Decreased S100A7 expression is linked to altered differentiation-, autophagy- and senescence-related programs during skin aging

**DOI:** 10.1038/s41514-026-00330-8

**Published:** 2026-01-17

**Authors:** Ge Peng, Fumihiro Hattori, Hideoki Ogawa, Ko Okumura, François Niyonsaba

**Affiliations:** 1 Atopy (Allergy) Research Center, Juntendo University Graduate School of Medicine, Tokyo, Japan; 2 Mikimoto Pharmaceutical, Mie, Japan; 3 Faculty of International Liberal Arts, Juntendo University, Tokyo, Japan

**Keywords:** Cell biology, Diseases

## Abstract

Skin aging involves progressive structural and functional decline, yet the underlying molecular mechanisms remain unclear. Here, we report that the antimicrobial peptide S100A7 is markedly reduced in aged keratinocytes and that its depletion leads to transcriptional alterations in differentiation-, autophagy-, and senescence-associated pathways. S100A7 knockdown partially recapitulated senescence-associated signatures, whereas supplementation increased autophagy and attenuated senescence-like phenotypes. These findings support a role for S100A7 as a context-dependent modulator of epidermal homeostasis and establish an AMP–autophagy axis that may contribute to cellular changes during skin aging.

## Introduction

The skin plays an essential role in shaping systemic physiology and barrier defense, yet it undergoes progressive structural and functional changes with age^1^. Increasing evidence suggests that the skin microbiome changes during aging in a way that reflects the underlying cutaneous biology^2–4^. However, the molecular mechanisms linking intrinsic epidermal changes with age-related loss of barrier function remain incompletely defined. Antimicrobial peptides (AMPs), produced predominantly by keratinocytes, are key regulators of cutaneous immunity^5^, but their involvement in aging has not been well characterized. Among these AMPs, S100A7 is notable not only for its antimicrobial functions but also for its broader roles in regulating epithelial differentiation, modulating oxidative stress, and participating in calcium-binding–dependent signaling pathways^6–8^. These multifunctional properties suggest that alterations in S100A7 expression may meaningfully influence epidermal homeostasis during skin aging.

**Fig. 1:**
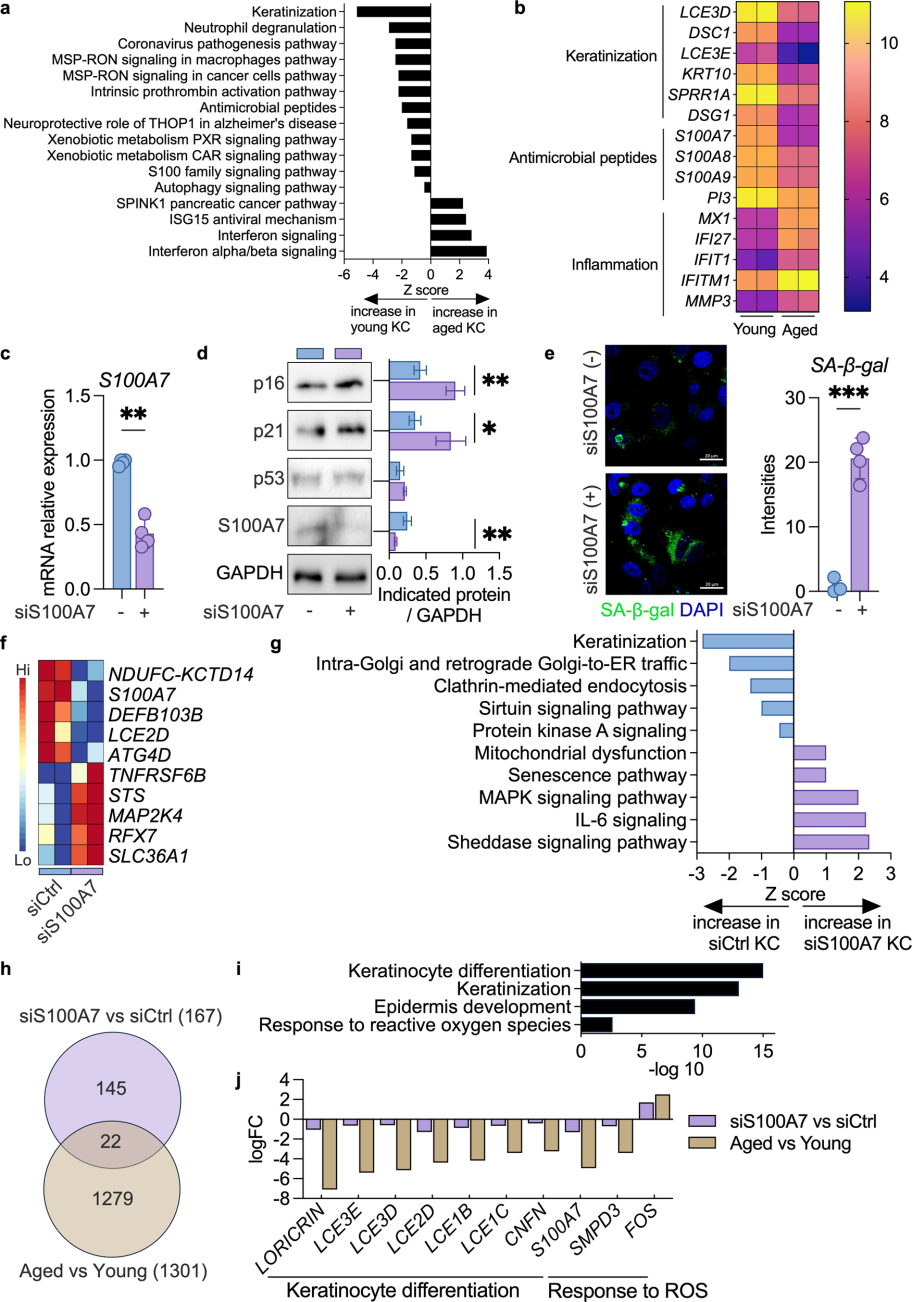
Downregulation of S100A7 is associated with keratinocyte aging phenotypes. **a** Ingenuity pathway analysis of keratinocytes derived from the back skin of two donors of the same sex and ethnicity but differing in age by 55 years (GSE108674). Pathways that are predicted to be upregulated or downregulated are shown with Z scores calculated on the basis of overall changes in gene expression. **b** Transcriptomic profiling of representative keratinization-, antimicrobial peptide-, and inflammation-related genes in young versus aged KCs. **c** Real-time PCR analysis of the *S100A7* gene showing the knockdown efficiency of *S100A7* in young keratinocytes. **d** Western blot analyses of the indicated proteins in S100A7-silenced and control young cells. GAPDH was used as a loading control. Full-length, uncropped blots with molecular weight markers are provided in Figure S2. **e** Representative SA-β-gal staining images (left) and quantification of SA-β-gal intensities in S100A7-silenced and control young cells (right). Scale bars: 10 μm. **f** Representative differentially expressed genes from RNA-seq analysis of young cells with or without S100A7 knockdown. The genes shown were manually selected on the basis of their known biological relevance to mitochondrial function, AMP expression, epidermal differentiation, autophagy and inflammation. **g** Ingenuity pathway analysis revealed several signaling pathways that were altered in S100A7-silenced or control young keratinocytes (KCs). Pathways predicted to be upregulated or downregulated are shown with Z scores calculated on the basis of overall changes in gene expression. **h** Venn diagram of DEGs regulated by both S100A7 knockdown and chronological aging. **i** Functional classification of common genes in (**h**) showing the involved pathways. **j** The top 10 genes whose expression was repressed upon S100A7 knockdown and aging in KCs. Underlined transcripts are known to be involved in differentiation or the response to reactive oxygen species (ROS). The fold change is from the transcriptome array analysis. *p < 0.05, ** *p* < 0.01, *** *p* < 0.001, **** *p* < 0.0001.

To explore age-related molecular alterations in keratinocytes, we analyzed the public GSE108674 dataset and compared the transcriptomes of keratinocytes derived from the back skin of two donors who were of the same sex and ethnicity but differed in age by 55 years. Pathway analysis revealed robust downregulation of keratinization and autophagy and increased inflammatory signaling with age (Fig. 1a). Notably, among the AMPs, the expression levels of members of the S100 family were significantly reduced, and S100A7 emerged as the most downregulated gene (Fig. 1b). A human protein atlas confirmed that S100A7 is more highly expressed in human skin than in other tissues (Figure S1a). Immunostaining demonstrated high S100A7 expression in the suprabasal layers of the epidermis, and this immunostaining was reduced in aged skin (Figure S1b). Because epidermal thinning accompanies chronological aging, reduced staining intensity may partially reflect decreased suprabasal cell numbers. We therefore further examined normal human epidermal keratinocytes (NHEKs) from donors of different ages (65 years). These donor-derived keratinocytes also presented decreased S100A7 transcript levels (Figure S1c), supporting a cell-intrinsic reduction independent of epidermal thickness. Together, these findings suggest a potential role for S100A7 in epidermal differentiation and aging.

To investigate whether reduced S100A7 contributes to keratinocyte aging, we silenced S100A7 in NHEKs and confirmed its efficient knockdown at both the transcript (Fig. 1c) and protein levels (Fig. 1d). S100A7 depletion notably induced senescence-like phenotypes^1^, including increased p16, p21 (Fig. 1d), and senescence-associated β-galactosidase (SA-β-gal) activity (Fig. 1e). Consistent with this, keratinocytes from aged donors expressed lower levels of cornified envelope genes and autophagy-related genes (*IVL*, *LORICRIN*, *MAP1LC3B*, and *BECN1*) and higher levels of senescence-associated secretory phenotype-related genes (*IL6* and *MMP3*), although these transcript measurements are presented descriptively without statistical analysis because only two biological donors were available per group (Figure S1c). Additionally, transcriptomic profiling of aged cells revealed decreased expression of representative epidermal differentiation and AMP-related genes (*KRT14*, *S100A7*, *DEFB103B*, *IVL*, and *LORICRIN*) and increased expression of markers associated with senescence, oxidative stress, and extracellular matrix remodeling (*SLFN11*, *DDIAS*, *SPARC*, *ADAMTS4*, and *CGAS*) (Figure S1d). Enrichment analysis confirmed reduced keratinization, AMP expression, and autophagy with enhanced mismatch repair in aged keratinocytes (Figure S1e). These findings indicate that the changes in S100A7 expression that occur during skin aging are maintained in isolated keratinocytes in vitro.

Strikingly, S100A7 knockdown in keratinocytes partially recapitulated senescence-associated transcription in aged cells (Figure S1d), including the downregulation of representative genes involved in mitochondrial function, AMP expression, epidermal differentiation, and autophagy (*NDUFC-KCTD14*, *DEFB103B*, *LCE2D*, and *ATG4D*) and the upregulation of stress- and inflammation-related transcripts (*TNFRSF6B*, *STS*, *MAP2K4*, *RFX7*, and *SLC36A1*) (Fig. 1f). Further pathway enrichment indicated impaired keratinization and increased mitochondrial dysfunction and senescence signaling in S100A7-silenced cells (Fig. 1g). Overall, twenty-two genes were commonly regulated by both S100A7 knockdown and chronological aging (Fig. 1h). The functional classification of these shared genes revealed suppression of keratinocyte differentiation and keratinization and exacerbation of the reactive oxygen species response. Manual inspection revealed that seven of the top ten differentially expressed genes, including *LORICRIN* and *LCE3E*, were jointly downregulated (Fig. 1j). Collectively, these findings indicate that senescence-associated decreases in S100A7 contribute to keratinocyte senescence through cell-autonomous processes, particularly through the impairment of terminal differentiation.

**Fig. 2:**
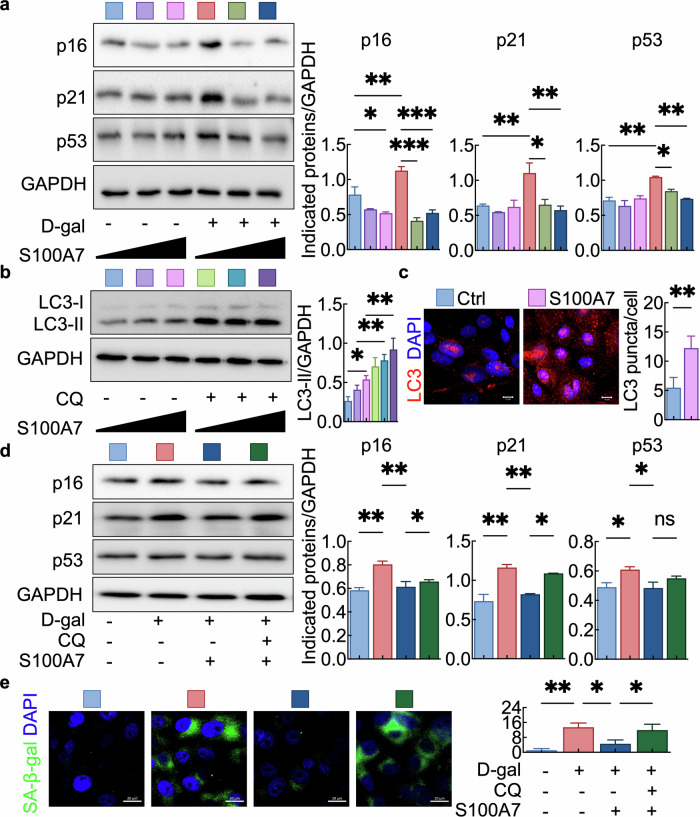
S100A7 supplementation reduces senescence in D-gal-induced keratinocyte aging via autophagy activation. **a** Young keratinocytes were stimulated for 24 hours with 10 or 50 ng/mL S100A7 in the presence (+) or absence (–) of 150 mM D-galactose (D-gal); *n* = 3 per group. Representative immunoblots of the indicated proteins (left) and quantification of band intensities (right). GAPDH was used as a loading control. Full-length, uncropped blots with molecular weight markers are provided in Figure S3a–d. **b** Young keratinocytes were stimulated for 24 h with 10 or 50 ng/mL S100A7 in the presence (+) or absence (–) of 10 μM chloroquine (CQ); *n* = 3 per group. Representative immunoblots of LC3 protein (left) and quantification of band intensities (right). GAPDH was used as a loading control. Full-length, uncropped blots with molecular weight markers are provided in Figure S3e, f. **c** Immunofluorescence analysis showing increased numbers of LC3-positive puncta in 50 ng/mL S100A7-treated young keratinocytes. Representative immunofluorescence images (left) and quantification of LC3 puncta in keratinocytes (right). Scale bars: 10 μm. (**d**) Young keratinocytes were stimulated for 24 h with 50 ng/mL S100A7 in the presence (+) or absence (–) of 150 mM D-gal or 10 μM CQ; *n* = 3 per group. Representative immunoblots of the indicated proteins (left) and quantification of band intensities (right). GAPDH was used as a loading control. Full-length, uncropped blots with molecular weight markers are provided in Figure S3g–j. **e** Young keratinocytes were stimulated for 24 hours with 50 ng/mL S100A7 in the presence (+) or absence (−) of 150 mM D-gal or 10 μM CQ; *n* = 3 per group. Representative SA-β-gal staining images (left) and quantification of SA-β-gal intensities (right). Scale bars: 10 μm. GAPDH was used as a loading control. *p < 0.05, ** *p* < 0.01, ***p < 0.001.

To evaluate whether S100A7 supplementation could mitigate senescence-like phenotypes, we used D-galactose (D-gal), a commonly used model that reproduces stress-driven senescence signatures in keratinocytes, to induce cellular aging^9,10^. D-gal-treated NHEKs exhibited senescence-like increases in p16, p21, and p53 expression, and these increases were markedly reduced by supplementation with 10 or 50 ng/ml S100A7 (Fig. 2a). Since both S100A7 and autophagy markers were downregulated in aged and S100A7-silenced keratinocytes (Figures S1c and 1f), we explored the mechanistic connection between S100A7 and autophagy markers. S100A7 supplementation increased autophagic flux, as reflected by elevated LC3-II levels in the absence of chloroquine. Notably, LC3-II also accumulates when cells are treated with S100A7 in the presence of chloroquine, which blocks autophagosome–lysosome fusion and prevents LC3-II degradation^11^. This pattern is consistent with S100A7 stimulating upstream autophagosome formation, whereas chloroquine inhibits the downstream degradative step, leading to LC3-II accumulation (Fig. 2b). In parallel, S100A7-treated cells presented increased numbers of LC3-positive puncta (Fig. 2c), further supporting enhanced autophagosome formation. Importantly, the suppression of D-gal-induced senescence-like markers, including p16, p21, and p53, as well as SA-β-gal, which was observed after treatment of the cells with 50 ng/ml S100A7, was abolished when autophagy was inhibited by chloroquine (Fig. 2d, e), demonstrating that the antiaging effect of S100A7 requires the activation of autophagy.

Collectively, our findings indicate that reduced S100A7 expression contributes to alterations in differentiation-, autophagy-, and senescence-associated transcriptional programs during keratinocyte aging. While S100A7 silencing led to decreased expression of canonical differentiation markers and impaired autophagic activity, these observations are based on transcriptomic signatures and cellular phenotypes rather than dedicated functional differentiation assays. Thus, our data support a model in which age-associated loss of S100A7 influences keratinocyte states but does not directly impair functional differentiation. Previous work has shown that S100A7 enhances keratinocyte differentiation marker expression and strengthens tight junction barrier integrity^12^, supporting the biological plausibility of our observations. Nevertheless, confirming a causal role for S100A7 in differentiation will require targeted assays such as Ca²⁺-induced differentiation kinetics or cornified envelope formation analyses.

S100A7 is detectable on the skin surface at 5–20 ng/cm² and at concentrations of up to 100 ng/cm² in sebaceous-rich areas^7^. Although measurements of its surface density on the skin are not directly equivalent to soluble concentrations in culture, our chosen range of 10–50 ng/ml approximates the estimated physiological levels within the epidermal milieu. Exposure to these concentrations of S100A7 was sufficient to restore autophagy and reduce senescence, whereas excessively high levels of S100A7, as observed in inflamed skin, may promote proinflammatory responses^6,8^. Thus, S100A7 emerges as a context-sensitive modulator of the AMP–autophagy axis. Its dual nature highlights both its importance for epidermal homeostasis and the need for caution when therapeutic approaches targeting S100A7 in skin aging are considered.

Although our data demonstrate that S100A7 supplementation enhances autophagic flux in keratinocytes, the upstream signaling events remain incompletely defined. S100A7 has multiple biochemical activities, including calcium binding, redox modulation, and interactions with advanced glycosylation end-product-specific receptors and other S100 family receptors^6^, all of which can influence autophagy-related pathways^13^. Given that oxidative stress is a key regulator of autophagy and senescence^13^, the possibility that S100A7 exerts its effects through the modulation of reactive oxygen species or Ca²⁺-dependent signaling cannot be excluded. Future mechanistic studies will be needed to elucidate these potential pathways.

Importantly, S100A7 has a dual and context-dependent nature. Under physiological conditions, S100A7 contributes to epidermal differentiation, barrier homeostasis, and protection against microbial stress^7,12^. In contrast, when highly induced in inflammatory skin disorders, such as psoriasis and atopic dermatitis, S100A7 can act as a proinflammatory amplifier downstream of IL-17, IL-22, and TNF-α signaling^6–8^. This functional dichotomy mirrors its opposing expression patterns—reduced in chronological aging but strongly upregulated in inflammatory disease—and underscores that S100A7 biology cannot be interpreted outside its microenvironmental context. Thus, reduced S100A7 in aged keratinocytes likely represents an aging-specific regulatory program rather than a universal response across all cutaneous conditions.

Given this duality, therapeutic manipulation of S100A7 should be approached with caution. Although restoring physiological levels of S100A7 may theoretically promote age-associated epidermal decline, excessive modulation could trigger unintended proinflammatory consequences, particularly in individuals who are predisposed to inflammatory skin disease. Future translational studies will need to carefully consider these context-dependent effects.

This work has several limitations. The transcriptome comparisons were based on a small number of donors, and the in vitro findings require validation in larger human cohorts and in vivo models. Furthermore, whether S100A7 interacts with other S100 family members or microbiome-derived signals during aging remains to be clarified. Future studies that combine multiomics analyses, organotypic skin models, and longitudinal clinical sampling may help address these gaps and explore translational opportunities.

## Methods

### Reagents

The recombinant human S100A7 used in this study was provided by R&D Systems, Inc. (Minneapolis, MN). D-Galactose was purchased from Tokyo Chemical Industry (Tokyo, Japan). Chloroquine was obtained from Sigma‒Aldrich (Burlington, MA).

### Primary normal human epidermal keratinocytes

Primary normal human epidermal keratinocytes were purchased from Kurabo Industries (Osaka, Japan). The keratinocytes were isolated from the abdominal skin of young (*n* = 2, male, 12 and 14 years old) and aged (*n* = 2, male, 77 and 79 years old) donors and were cultured in serum-free HuMedia-KG2 keratinocyte growth medium (Kurabo Industries) supplemented with human epidermal growth factor (0.1 ng/mL), insulin (10 μg/mL), hydrocortisone (0.5 μg/mL), gentamicin (50 μg/mL), amphotericin B (50 ng/mL), and bovine brain pituitary extract (0.4%, vol/vol) at 37 °C in a humidified atmosphere of 95% air and 5% CO_2_ as previously described^14^. Cells that had reached 80% confluence were continually cultured in medium containing high concentrations (1.8 mM) of Ca^2+^ for 24 h to mimic keratinocytes of the suprabasal layer^15^.

### Transfection with siRNA

Young cultured cells were transfected with 30 pmol of the siRNA duplex targeting *S100A7* (Thermo Fisher, Waltham, MA) or with scrambled control siRNA (Invitrogen, Waltham, MA) using Lipofectamine RNAiMAX (Invitrogen) for 24 h according to the manufacturer’s directions. The transfection efficiency was evaluated via real-time quantitative PCR.

### Real-time quantitative PCR

Total RNA was extracted from keratinocytes using an RNeasy Plus Micro Kit (Qiagen, Hilden, Germany). To generate first-strand cDNA, reverse transcription of 1 μg of total RNA was performed using ReverTra Ace qPCR RT Master Mix (Toyobo, Osaka, Japan) according to the manufacturer’s instructions. Real-time PCR was performed using the QuantiTect SYBR Green PCR Kit (Qiagen). Amplification and detection of mRNA were performed using the StepOnePlus Real-Time PCR System (Life Technologies, Waltham, MA) according to the manufacturer’s instructions. The sequence-specific primer sets used in this study are listed in Table S1. All real-time PCRs were performed in triplicate, and fold changes in gene expression are reported relative to the values in the untreated controls.

### Western blotting

Human keratinocytes and mouse skin tissues were lysed in RIPA lysis buffer (Cell Signaling Technology). Protein concentrations were determined using Precision Red Advanced Protein Assay reagent (Cytoskeleton, Denver, CO), and equal amounts of total protein were subjected to electrophoresis on 8–15% SDS‒PAGE gels, followed by transfer to PVDF membranes (Merck Millipore, Burlington, MA). The membranes were then blocked in ImmunoBlock buffer for 1 hour at room temperature, followed by overnight incubation at 4 °C with primary antibodies according to the manufacturer’s instructions. The primary antibodies were detected using appropriate secondary antibodies, developed with Luminata Forte Western horseradish peroxidase substrate (Merck Millipore, Billerica, MA), and imaged using Fujifilm LAS-4000 Plus. ImageJ was used to quantify the intensities of the bands in the images. The antibodies used in this study are listed in Table S2.

### SA-β-gal staining

SA-β-gal activity was assessed using a Cellular Senescence Detection Kit-SPiDER-βGal (Dojindo, Kumamoto, Japan), a fluorogenic probe–based method that detects increased β-gal activity in senescent cells with high sensitivity^16^. Staining was performed according to the manufacturer’s protocol, and SA-β-gal-positive cells exhibited stronger fluorescence under a confocal laser scanning microscope (Ex: 488 nm; Em: 500–600 nm). The fluorescence intensity was quantified using ImageJ (NIH, Bethesda, MD).

### Immunocytochemistry analysis

Keratinocytes were plated on 12-mm-diameter coverslips. After the indicated treatments, the coverslips to which the cells were attached were fixed in preheated 4% paraformaldehyde in PBS for 10 minutes, quenched with NH_4_Cl for 10 min, permeabilized with 0.01% Triton X-100 in PBS for 5 min, blocked with ImmunoBlock at 4 °C for 30 min, and incubated overnight at 4 °C with an antibody against LC3. After incubation, the cells were stained with an Alexa Fluor 594-conjugated goat anti-rabbit antibody. Images were analyzed on a Zeiss Laser Scanning Microscope 780 system, and quantification of the fluorescence intensities of the images was performed with ImageJ. The antibodies used in this study are listed in Table S2.

### Bulk RNA sequencing

Total RNA was isolated from cultured NHEKs using an RNeasy Plus Micro Kit according to the manufacturer’s instructions. RNA quantity and purity were assessed by NanoDrop spectrophotometry, and RNA integrity was confirmed using an Agilent 4200 TapeStation with High-Sensitivity RNA ScreenTape (Agilent Technologies, Santa Clara, CA). Libraries were prepared using the TruSeq Stranded mRNA Library Prep Kit (Illumina, San Diego, CA); the process included poly(A) mRNA enrichment and fragmentation followed by cDNA synthesis, end repair, adaptor ligation, and PCR amplification. Sequencing was performed on the Illumina NovaSeq 6000 platform, generating 150-bp paired-end reads. The quality of the raw reads was checked using FastQC, and low-quality bases and adaptors were trimmed using Trimmomatic. Clean reads were aligned to the human reference genome (GRCh38) using STAR aligner, and gene-level counts were quantified with featureCounts. Differential gene expression analysis was conducted using edgeR; changes in expression were considered significant at adjusted *P* < 0.05 and |log2-fold change | ≥ 1. The bulk RNA sequencing data were subsequently analyzed to identify the affected signaling pathways using Ingenuity Pathway Analysis (Ingenuity Systems, IPA Winter 2020 series).

### Statistics

All the statistical analyses were performed with GraphPad Prism 10 software (version 10.1.1). Two-tailed Student’s t test was used to compare 2 groups, and one-way ANOVA with Tukey’s multiple-comparison test was used for comparisons of multiple groups. *P*-values less than 0.05 were considered to indicate statistical significance.

## Supplementary information

Supplementary information

## Data Availability

The data that support the findings of this study are openly available in the Gene Expression Omnibus at https://www.ncbi.nlm.nih.gov/geo, reference number GSE312362.
