# Supplementary material for: Decreased S100A7 expression is linked to altered differentiation-, autophagy- and senescence-related programs during skin aging
Source: NPJ Aging. 2026 Jan 17;12(1):31. doi: 10.1038/s41514-026-00330-8 (PMC12916949; doi:10.1038/s41514-026-00330-8)
Supplement: Supplementary file 1 — Supplementary information [file 41514_2026_330_MOESM1_ESM.pdf]

Supplementary materials

Decreased S100A7 expression is linked to altered differentiation-, autophagy- and senescence-related programs during skin aging

Ge Peng [1, *, #], Fumihiro Hattori [2, *], Hideoki Ogawa [1], Ko Okumura [1], François Niyonsaba [1, 3, #]

[1] Atopy (Allergy) Research Center, Juntendo University Graduate School of Medicine, Tokyo, Japan

[2] Mikimoto Pharmaceutical, Mie, Japan

[3] Faculty of International Liberal Arts, Juntendo University, Tokyo, Japan

* Co-first authors, # Cocorresponding authors

Corresponding author:

Ge Peng, MD, PhD

Atopy (Allergy) Research Center, Juntendo University Graduate School of Medicine

2-1-1 Hongo, Bunkyo-Ku, Tokyo 113-8421, Japan

Email: g-peng@juntendo.ac.jp

François Niyonsaba, MD, PhD

Atopy (Allergy) Research Center, Juntendo University Graduate School of Medicine

2-1-1 Hongo, Bunkyo-Ku, Tokyo 113-8421, Japan

Email: francois@juntendo.ac.jp

Figure S1

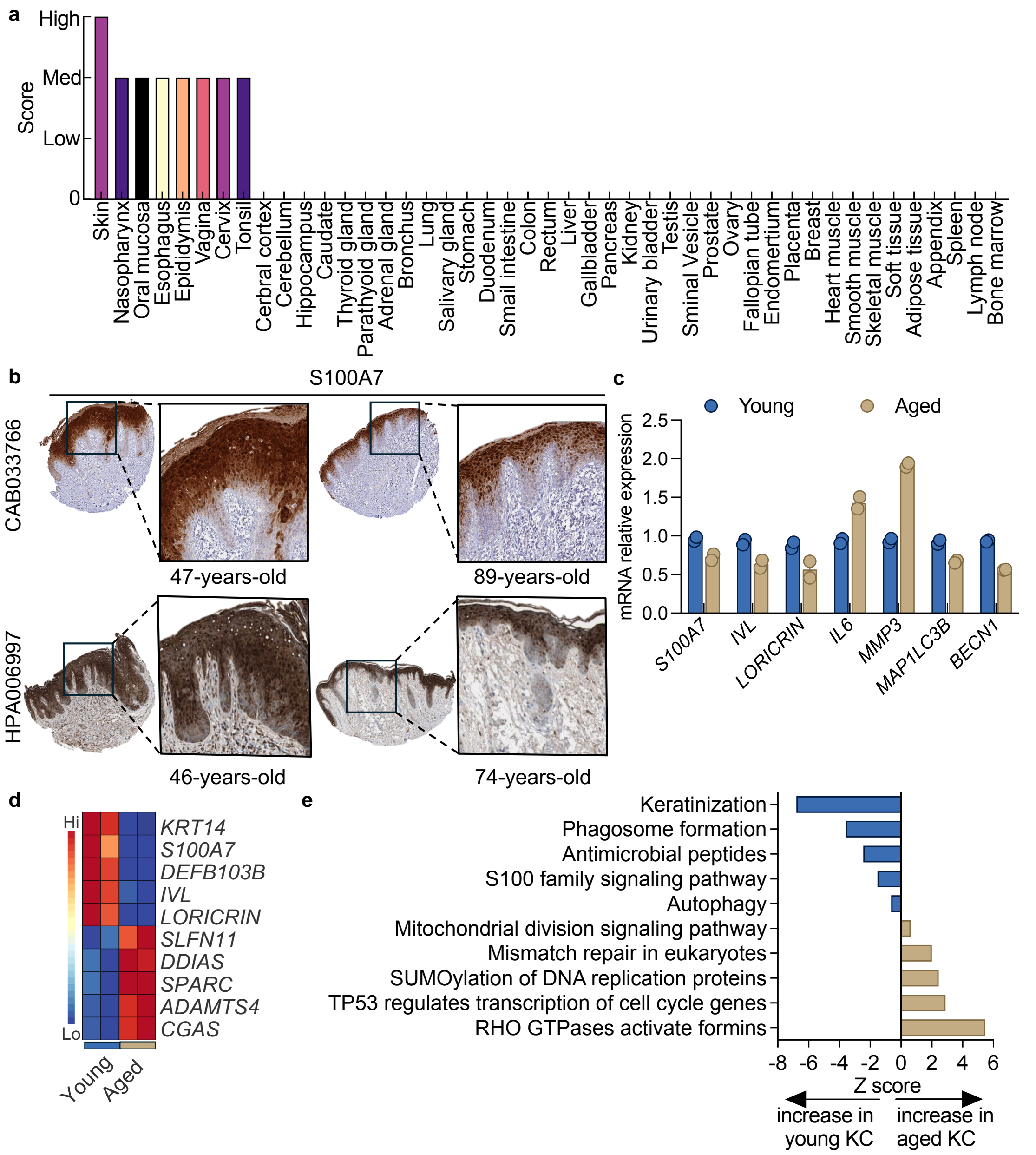

Figure S1. S100A7 expression is decreased in human epidermis from aged individuals

(**a**) Protein expression levels of S100A7 in 44 human tissues reported in the Human Protein Atlas (https://www.proteinatlas.org/ENSG00000143556-S100A7/tissue). (**b**) Representative immunohistochemistry images showing S100A7 expression in human skin from young (46–47 years) and aged (74–89 years) donors stained with two

independent antibodies (CAB033766 and HPA006997) obtained from the Human Protein Atlas (https://www.proteinatlas.org/ENSG00000143556-S100A7/tissue/skin). (**c**) Real-time PCR analysis of the indicated genes in cells from young (n=2) versus aged (n=2) donors (65-year difference). Because biological replicates are limited, no statistical testing was performed, and the data were interpreted descriptively. (**d**) Representative differentially expressed genes from RNA-seq analysis of young (12 and 14 years old) and aged (77 and 79 years old) keratinocytes. The genes shown were manually selected on the basis of their known biological relevance to keratinocyte differentiation, AMP expression, oxidative stress, extracellular matrix remodeling, and senescence. (**e**) Ingenuity pathway analysis revealed several signaling pathways that were altered in young or aged keratinocytes (KCs). Pathways predicted to be upregulated or downregulated are shown with Z scores calculated on the basis of overall changes in gene expression.

Figure S2

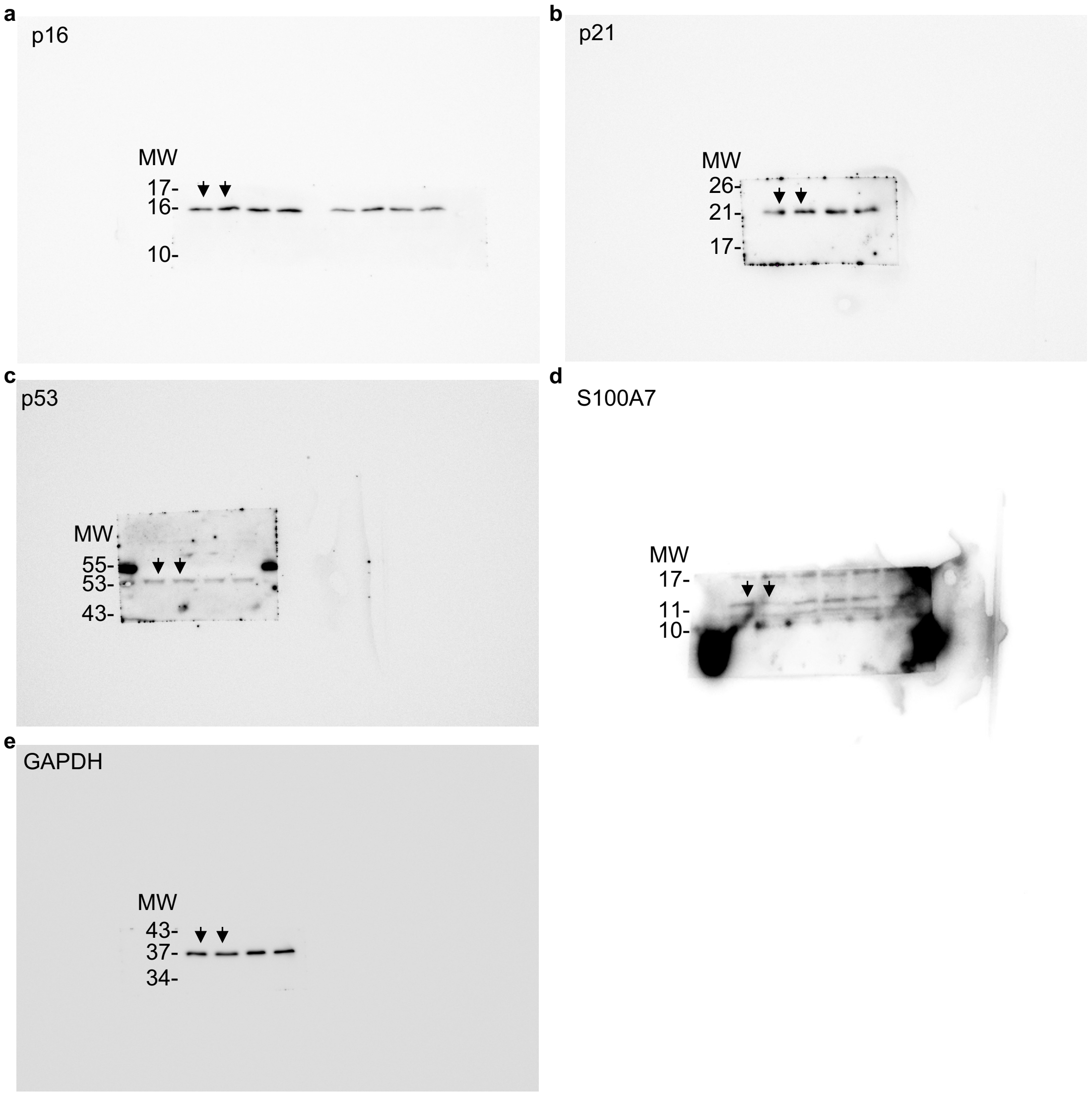

Figure S2. Uncropped and unprocessed blots corresponding to Figure 1d.

Full-length, uncropped blots for p16 (**a**), p21 (**b**), p53 (**c**), S100A7 (**d**), and GAPDH (**e**) shown in Figure 1d. The molecular weight (MW) markers are indicated on the left side of each blot. Arrows highlight the specific regions used in the cropped images shown in Figure 1d. All scans were obtained directly from the original blot images without any contrast or gamma adjustments.

Figure S3

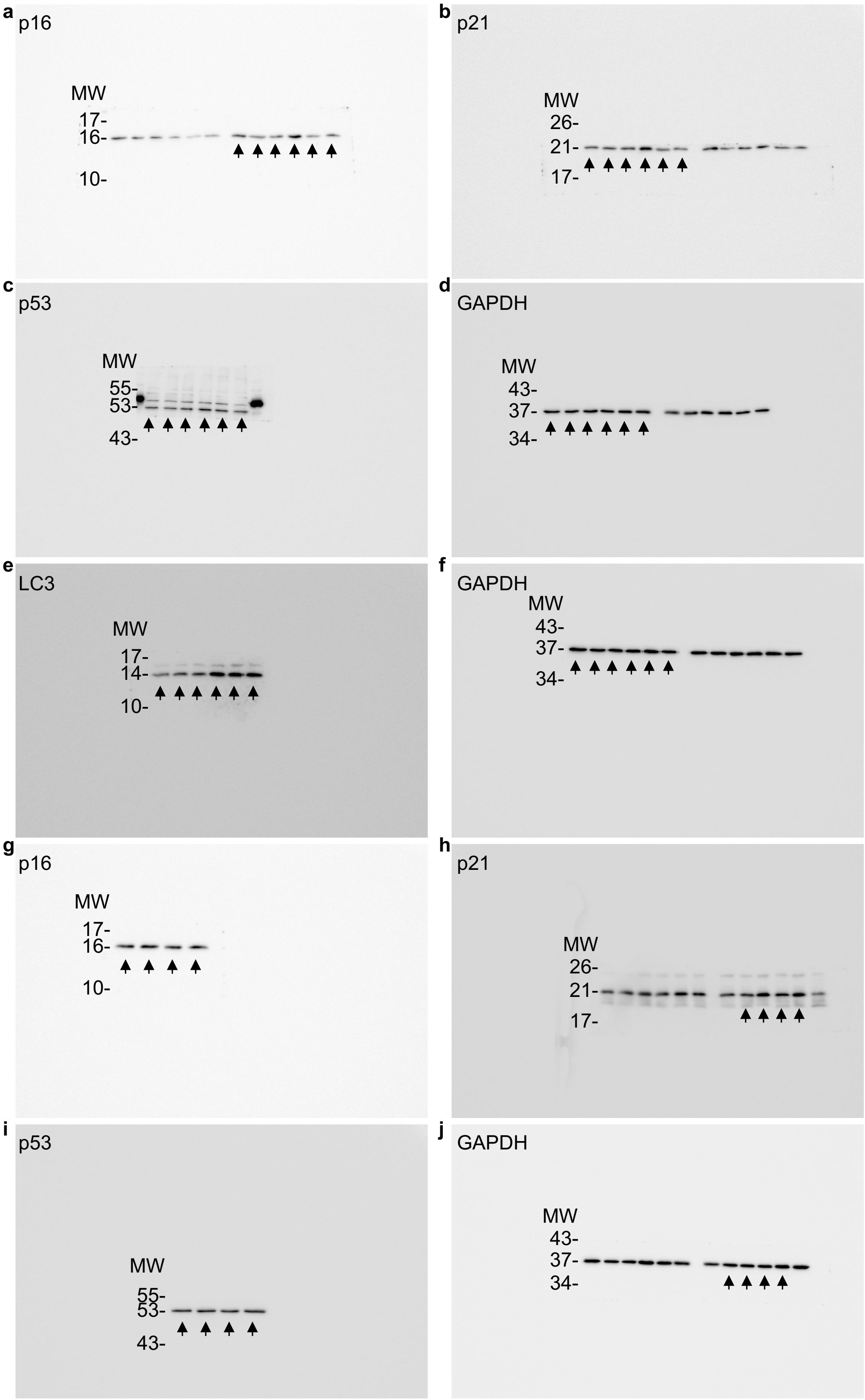
a
p16
MW
17-
16-
10-
b
p21
MW
26-
21-
17-
c
p53
MW
55-
53-
43-
d
GAPDH
MW
43-
37-
34-
e
LC3
MW
17-
14-
10-
f
GAPDH
MW
43-
37-
34-
g
p16
MW
17-
16-
10-
h
p21
MW
26-
21-
17-
i
p53
MW
55-
53-
43-
j
GAPDH
MW
43-
37-
34-

Figure S3. Uncropped and unprocessed blots corresponding to Figure 2.

Full-length blots for p16 (**a**), p21 (**b**), p53 (**c**), and GAPDH (**d**) corresponding to Figure 2a; LC3 (**e**) and GAPDH (**f**) corresponding to Figure 2b; and p16 (**g**), p21 (**h**), p53 (**i**), and GAPDH (**j**) corresponding to Figure 2d are shown. The molecular weight (MW) markers are indicated on the left side of each blot. Arrows highlight the specific regions used in the cropped images shown in Figure 2. All scans were obtained directly from the original blot images without any contrast or gamma adjustments.

Table S1. Sequences of the primers used in real-time PCR

Primer name	Primer sequence (5'- to -3')
hRPS18	
F	TTTGCGAGTACTCAACACCAACATC
R	GAGCATATCTTCGGCCCACAC
S100A7	
F	CACCAGACGTGATGACAAGATTGA
R	AGACATCGGCGAGGTAATTTGTG
IVL	
F	TTCTAAGATGTCCCAGCAACACAC
R	GTTTCATTTGCTCCTGATGGGTA
LORICRIN	
F	GGCTGCATCTAGTTCTGCTGTTTA
R	CAAATTTATTGACTGAGGCACTGG
IL6	
F	AGACAGCCACTCACCTCTTCAG
R	TTCTGCCAGTGCCTCTTTGCTG
MMP3	
F	GGGTGAGGACACCAGCATGA
R	CAGAGTGTCGGAGTCCAGCTTC
MAP1LC3B	
F	GATGTCCGACTTATTCGAGAGC
R	TTGAGCTGTAAGCGCCTTCTA
BECN1	
F	AGCTGCCGTTATACTGTTCTG
R	ACTGCCTCCTGTGTCTTCAATCTT

Table S2. List of antibodies used in this study

Primary antibodies			
**Antibodies**	**Catalog No.**	**Dilution**	**Company**
p16	80772	1:1000	Cell Signaling Technology, Beverly, MA
p21	2947	1:1000	Cell Signaling Technology, Beverly, MA
p53	9282	1:1000	Cell Signaling Technology, Beverly, MA
S100A7	26656-1-AP	1:1000	Proteintech, Rosemont, IL
GAPDH	60004-1-lg	1:100000	Proteintech, Rosemont, IL
LC3	PM036	1:1000	MBL, Nagoya, Japan
**Secondary antibodies**			
Alexa Fluor 594 goat anti-rabbit IgG (H+L)	A11037	1:1000	Invitrogen, Waltham, MA
Sheep anti-rabbit antibody conjugated to HRP	NA934V	1:5000	Cytiva, Marlborough, MA
Sheep anti-mouse antibody conjugated to HRP	NA931V	1:5000	Cytiva, Marlborough, MA
